# Evaluation of Bio-Rejuvenator and Compaction Conditions on Stiffness Modulus and Indirect Tensile Strength of Recycled Hot Mix Asphalt

**DOI:** 10.3390/ma17133081

**Published:** 2024-06-22

**Authors:** Andrei Forton, Adrian Ciutina, Adelin Stirb, Paul Marc, Ciprian Costescu, Alexandra Ciopec

**Affiliations:** Department of Overland Communication Ways, Foundation and Cadastral Survey, University Politehnica Timisoara, 1A Ioan Curea, 300224 Timisoara, Romania; adrian.ciutina@upt.ro (A.C.); adelin.stirb@upt.ro (A.S.); paul.marc@upt.ro (P.M.); ciprian.costescu@upt.ro (C.C.); alexandra.ciopec@upt.ro (A.C.)

**Keywords:** RAP material, rejuvenator, stiffness modulus, indirect tensile strength, workability, compaction

## Abstract

This study focuses on the investigation of the effect of a reclaimed asphalt material (RAP) and a bio-rejuvenator (mix of vegetable oils) on the stiffness modulus and indirect tensile strength (ITS) values of eight bituminous mixtures produced by using three types of compaction, with different RAP amounts (25% and 50%) and rejuvenator (0%, 0.20%, 0.40% and 0.60% by mass of RAP). A conventional hot mix asphalt was considered as the reference mix. All tests were performed on cylindrical samples produced using: Marshall compaction with 50 blows/side, cored cylindrical specimens from slabs compacted using a roller compactor (39 passes), and, respectively, gyratory compaction on 80 gyrations. Stiffness modulus and ITS values showed strong linear variation with the increase in rejuvenator content, independently of test temperature and type of compaction. The rejuvenating effect of the bio-rejuvenator was observed to counterbalance the impact of RAP. The results at 20 °C for gyratory specimens for the mix with 50% RAP and 0.40% bio-rejuvenator were comparable/closer (under 5% relative difference) to those obtained for the reference mix. A strong correlation between stiffness modulus values of mixes and penetration values of the corresponding binder blends was obtained ($R^{2}\geq 0.977$).

## 1. Introduction

In order to fulfill the sustainable development principles, many road administrations across the globe over the past decades have invested in multiple research projects concerning two aspects: (i) how to better reuse and recycle the reclaimed asphalt pavement materials (RAP) and (ii) how to increase the RAP content within new bituminous mixtures [1,2,3,4]. Various procedures and technologies concerning these aspects were proposed and applied successfully, e.g., the use of rejuvenators, additives and other industrial waste materials in the production of warm mix asphalt (WMA) and hot mix asphalt (HMA) with high amounts of RAP [1,2,3,4,5,6,7,8].

Many studies have been carried out to investigate, analyze and describe the impact of different rejuvenators on the behavior of the RAP binder and on the performance properties of bituminous mixtures produced with RAP materials. Nowadays, various products are used as rejuvenators or recycling agents, such as mahua oil [9], softening agents, aromatics, tall oil, fluxing agents (flux oil, slurry oil, etc.), waste engine oil [10], soybean oil [11], composite castor oil [12], food waste bio-oil [13], oleic acid, naphthenic oil and paraffinic oil [14], and bio-oil additives [15,16,17,18]. The impact of bio-rejuvenators (mixes of vegetable origin) on the behavior of bituminous mixtures produced with RAP materials is an important topic that is gaining more attention considering the sustainable development requirements of the asphalt industry.

Several researchers showed that these rejuvenators can have the ability to counterbalance the effect of the aged RAP binder and provide a blend with thermomechanical behavior similar to that of a ‘fresh’ binder [19,20,21,22,23,24,25,26,27]. A true rejuvenator should present the capability to restore the properties of the aged binder in terms of rheological, thermomechanical and chemical characteristics [18,28,29].

Due to the fact that the rejuvenation process for rejuvenators and binders is a complex mechanism, at this point, there is no unified standard to evaluate its rejuvenation effect [30]. Consequently, extensive testing procedures are required when using rejuvenators during the production of asphalt mixes in order to quantify their impact on the final mix performance and also, to determine the proper dose and its diffusion and dispersion in the mix in terms of macro mechanical and micromechanical characterization and chemical composition.

Regarding a bituminous mixture’s behavior, it is well known that when significant amounts of a reclaimed asphalt material are used, due to the hardening effect of its aged binder, the final mix will become stiffer and more susceptible to cracking, affecting also its workability [1,31]. Therefore, rejuvenators are recommended to be used in order to prevent the mentioned problems and to improve the performance properties of the final mix.

Various studies highlight that an optimum use of rejuvenators improves the behavior of the final mix by lowering the cracking potential and by improving the short-term performance [32,33,34], the permanent deformation of the final mix [2,35] and the rutting resistance [1,8,34]. Moreover, they can reduce the stiffness [8,32,34,35] and moisture susceptibility compared to the mixtures made with virgin materials [8,34,36,37], and they can improve the low temperature cracking susceptibility [38,39]. However, according to the National Road Research Alliance (NRRA) report from 2020, rejuvenators may have a negative impact on the long-term performance, such as stability, rutting resistance, and moisture damage, under certain exposure conditions, such as high temperatures, for a long period of time [39].

Another important aspect related to the use of rejuvenators is that they can increase the flexibility of mixtures and improve their production process as well as the compacting process with minimum application of energy, without compromising their workability, performance and environmental impact [8,40,41]. If the rejuvenator dose is not optimal, it impacts the workability of bituminous mixtures produced with RAP material; therefore, their compaction is negatively impacted, which will lead to mixes more susceptible to cracking, undesired fatigue resistance and various other stresses that directly affect their durability.

Hence, the type and optimal dose of the rejuvenator have a fundamental influence on the behavior both of bitumen blends, by reducing the performance grade, increasing the penetration values, and decreasing the softening point values, and of mixes produced with RAP binder/material, by offering better cracking performance, longer fatigue life, decreased stiffness, and better workability [42,43,44].

The main goal of this study was to investigate the effect of a RAP material, obtained from an existing mixture for a national road in Timis county, Romania, and of a rejuvenator of vegetable origin (a mixture of vegetable oils) on the stiffness modulus and water sensitivity of eight asphalt mixes produced with various doses of RAP and with and without rejuvenator: eight HMAs containing 25% and 50% RAP and four rejuvenator doses, of 0%, 0.20%, 0.40% and 0.60% by mass of the RAP material. Also, a conventional HMA, produced only with virgin material, was considered as a reference mix. All mixes were designed to have a continuous 16 mm grading curve, identical total bitumen content of 5.60%—rejuvenator not included—as well as a grading curve closer to the one used for the reference mix.

Two types of tests, namely indirect tension tests on cylindrical specimens (IT-CY) performed at four temperatures of 10 °C, 15 °C, 20 °C and 25 °C, and indirect tensile strength (ITS) tests at 20 °C, together with volumetric measurements, were performed. These tests were performed on samples with a diameter of 100 mm, produced by using three types of compaction: cylindrical specimens produced by using Marshall compaction of 50 blows/each side; cored cylindrical specimens from slabs compacted in the lab by using a steel roller compactor considering the same level of compaction for all mixes: 39 passes; and cylindrical specimens produced by using gyratory compaction of 80 gyrations. Additionally, the same compaction energy for each type of compaction was used in all mixes, thus concentrating on the impact of the rejuvenator on the stiffness modulus and on the indirect tensile strength test results.

Finally, possible correlations between the stiffness modulus of mixes and the penetration values of the corresponding binder blends and the indirect tensile strength test results were investigated.

## 2. Materials and Methods

In this study, nine types of bituminous mixtures with the maximum aggregate size of 16 mm were produced and tested. All materials used in the production of these bituminous mixtures were chosen according to the Romanian norms and legislation. Quarry crushed aggregates (0–4 mm, 4–8 mm and 8–16 mm), natural sand 0–4 mm (used for five bituminous mixtures), limestone filler, RAP material 0–8 mm and 8–22.4 mm (used for eight bituminous mixtures), a 50/70 binder that is one of the most common binders used in Romania, and a rejuvenator of vegetable origin (used for six bituminous mixtures) were used in different doses to produce nine types of bituminous mixtures with and without RAP material and rejuvenator.

All materials used in the design and production of the analyzed mixes are common materials used in the road industry in Romania. As well, their proportions in the mass of the final mixes were chosen with respect to the Romanian norms. All fresh materials were independently investigated in a previous study [45], and the obtained experimental results were consistent with the Romanian norms and specifications used in the fabrication process of bituminous mixtures.

The characteristics of the RAP material were investigated by performing a series of laboratory tests according to the SR EN 13108-8 norm [46]. The results showed that the RAP material could be used in the production of new bituminous mixtures [45]. The RAP binder content in the analyzed RAP material was 4.0% [45]. In this step, a decision was made to divide the RAP material into two lots of 0–8 mm and 8–22.4 mm to improve the reuse of this material in the production of new mixtures. More details are given in Section 2.2.

### 2.1. Experimental Plan

Eight bituminous mixtures, all having a continuous 16 mm grading curve, the same total bitumen content of 5.60% (fresh bitumen + RAP bitumen, rejuvenator not included) and used in the same proportions for the RAP material (25% RAP 0–8 mm + 75% RAP 8–22.4 mm), were produced and tested in order to investigate the potential impact of the bio-rejuvenator, RAP and compaction type on their performance. The component proportions were determined in order to finally produce HMA mixes containing 25% and 50% RAP material and four doses of rejuvenator, of 0%, 0.20%, 0.40% and 0.60%, by mass of the RAP material. Section 2.2 offers more details regarding sample composition, design and production.

In order to have a basis of comparison, a conventional HMA was produced in this study, without RAP material and rejuvenator.

The bituminous mixtures were named as follows:

- ‘M’ for bituminous mixture;
- ‘0’, ‘25’ and ‘50’—the RAP material amount in percentage;
- ‘R’ for rejuvenator;
- ‘0’, ‘1’, ‘2’, ‘3’, which represent the dose of the rejuvenator, where ‘0’ corresponds to 0% rejuvenator, ‘1’ corresponds to 0.20% rejuvenator, ‘2’ corresponds to 0.40% rejuvenator, and ‘3’ corresponds to 0.60% rejuvenator by the mass of RAP material.

These nine types of bituminous mixes produced with these specific amounts of RAP and rejuvenator were chosen with respect to previous studies [20,22,23,24], where the corresponding bitumen blends were investigated in terms of thermomechanical characteristics.

**Figure 1:**
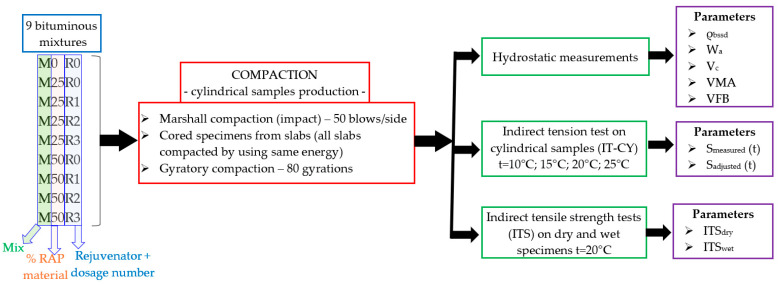
Experimental plan.

As presented in Figure 1, the experimental plan included hydrostatic volumetric measurements in order to determine the bulk density—saturated surface dry ($\rho_{\mathrm{bssd}}$), the water absorption ($W_{a}$), the void content ($V_{c}$), the voids in mixing aggregates (VMA) and the voids filled with bitumen (VFB); indirect tension tests on cylindrical specimens (IT-CY) performed at four temperatures; and indirect tensile strength tests on dry and wet specimens. The test procedures are briefly described in Section 2.4, Section 2.5 and Section 2.6. All four mentioned tests—volumetric measurements, IT-CY tests, and ITS dry and wet tests—were performed on all 9 types of bituminous mixtures, 3 samples for each type of compaction. A total of 36 cylindrical samples were produced and tested for each type of mixture.

### 2.2. Bituminous Mixture Production

The results for the conventional HMA reference mix (M0R0) that was studied and presented in a previous paper [45] were considered and used as a basis in this study.

In a second step, four bituminous mixtures containing 25% RAP material and 0%, 0.20%, 0.40% and 0.60% rejuvenator by mass of the RAP material were produced. These mixes were designed with a similar 5.60% total bitumen content (without the rejuvenator) and identical RAP material proportion 25% RAP 0–8 mm + 75% RAP 8–22.4 mm in order to obtain a grading curve similar/closer to the one used for the reference mix.

Thirdly, four other bituminous mixtures containing 50% RAP material and 0%, 0.20%, 0.40% and 0.60% rejuvenator by mass of the RAP material were designed by applying the same conditions as above.

The proportions of all base materials used in the production of the above-mentioned mixes are presented in Table 1.

**Figure 2:**
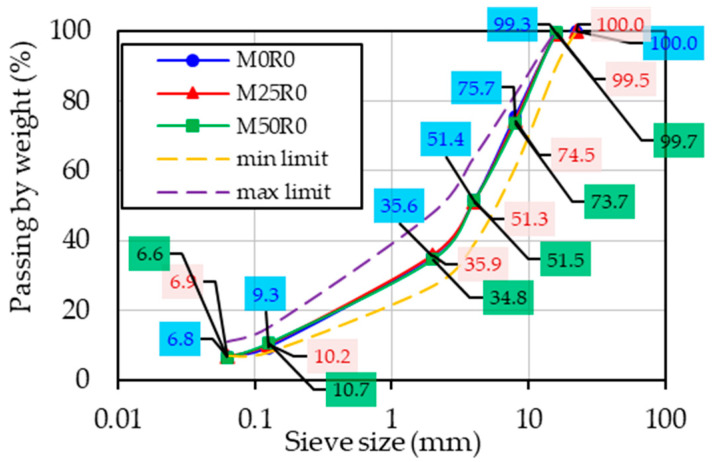
Bituminous mixture grading curves.

Figure 2 presents the grading curves used in the production phase of the analyzed mixes, together with the limits imposed by the Romanian norm AND 605 for bituminous mixtures with the maximum aggregate size of 16 mm. As an example, M50R1, M50R2 and M50R3 were designed by using the same grading curve as the one for M50R0. Similar observations apply in the case of the mixtures produced with 25% RAP material.

A heated mechanical mixer was used to carry out the mixing procedure, and the temperature was set at 160 ± 10 °C in accordance with AND 605 [47] and SR EN 12697-35 [48] standards.

Prior to the production stage, all materials were conditioned according to the normative specifications. Therefore, the virgin aggregates were preconditioned for 12 h at mixing temperature. The RAP material was conditioned at 165 ± 10 °C for 2 h, and the 50/70 bitumen was preheated at 160 ± 10 °C for 4 h before mixing. This was the order in which the base materials were introduced in the mixer. After this step, the rejuvenator at ambient temperature was introduced in the mixer. All base materials were mixed for a total of 5 min.

### 2.3. Specimen Fabrication

Three different types of compaction were used in this study:

- Marshall compaction or impact compaction by which cylindrical specimens with a diameter of 100 mm were compacted by using 50 blows/each side, according to SR EN 12697-30 [49]. A total of 108 Marshall samples were produced and tested;
- cored cylindrical specimens from slabs compacted in the laboratory by using a steel roller compactor according to SR EN 12697-33 [50]. A total of 18 slabs of 305 mm × 400 mm, two for each type of bituminous mixture, were compacted by using 25 kg weight of mixture and identical level of compaction of 39 passes, as follows: 2 passes with 0.50 kN compaction load, followed by 4 × 1.00 kN, 8 × 2.00 kN, 8 × 3.00 kN, 8 × 7.00 kN and 9 × 11.00 kN. After compaction, the slabs were kept for 10 h at ambient temperature, then each slab was demolded. All slabs were maintained for 72 h at 25 ± 5 °C before coring. From each slab, six cylindrical specimens with a diameter of 100 mm were obtained (a total of 108 cored samples). After the coring process, all samples were washed and kept for 72 h at ambient temperature before testing;
- gyratory compaction by which cylindrical specimens with a diameter of 100 mm were compacted according to SR EN 12697-31 [51] by using a constant number of 80 gyrations for all types of mixes, thus assuring similar constant compaction energy. For each sample compaction, similar conditions were applied: the load of 600 kPa, internal angle of 0.820°, speed of 30 rpm and compaction stop after 80 gyrations. Thus, a total of 108 gyratory samples were produced and tested.

Therefore, in this study, a similar compaction energy was used to produce three batches of samples, one for each compaction type independently of the bituminous mixture type or composition. The compaction energy was chosen with respect to the specification of AND 605 for a conventional HMA. A total of 324 cylindrical specimens with a diameter of 100 mm were produced and tested.

### 2.4. Hydrostatic Measurements

The volumetric characteristics of all the bituminous mixture specimens were determined by using the hydrostatic methods presented in the Romanian/European Standards SR EN 12697-6 [52], AND 605-Annex B [47] and SR EN 12697-8 [53] in order to finally obtain the bulk density—saturated surface dry ($\rho_{\mathrm{bssd}}$), the water absorption ($W_{a}$) and the void content ($V_{c}$, VMA and VFB).

It must be mentioned that the values for the maximum density of all bituminous mixtures, the amount of the binder and its density were the same as in the previous study [45].

A mean value of each previous mentioned parameter was obtained as the average of the three values obtained for three specimens of each mix. All results are reported in Section 3.

### 2.5. IT-CY Test

Indirect tension tests on cylindrical specimens (IT-CY) were performed on all bituminous mixtures, independently of the compaction type, according to the specifications of SR EN 12697-26 [54]. According to Romanian Norm AND 605, the stiffness modulus of a bituminous mixture should be checked in the lab, based on the test procedure described in SR EN 12697-26 [54], at a test temperature of 20 °C on cylindrical specimens prepared only by using the gyratory press.

Compared to the standard test procedure, some changes were made in this study regarding the specimen preparation and the test temperature. Three samples for each type of compaction/each type of bituminous mixture were tested at four temperatures: 10 °C, 15 °C, 20 °C and 25 °C, respectively. These conditions were selected to investigate how the test temperature affected the behavior of mixes as RAP and rejuvenator amounts increased.

Before testing, all specimens were conditioned at the test temperatures for four hours. After testing, all the samples were kept for 72 h at ambient temperature, and then the conditioning time started for the next test temperature.

**Figure 3:**
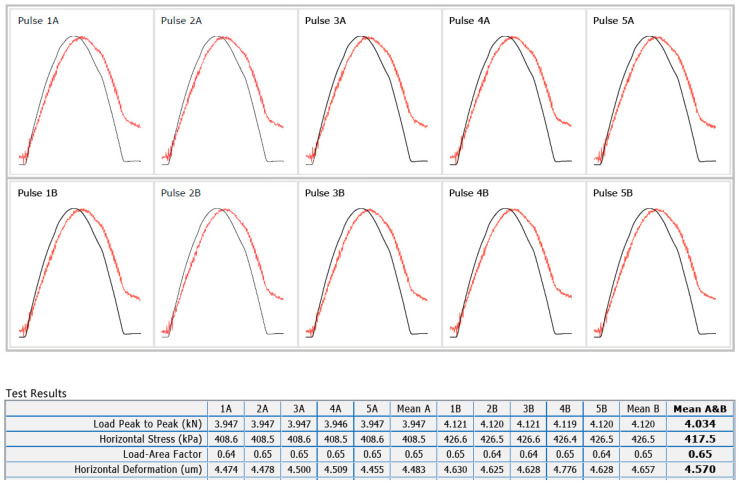
Test report for sample no. 2 (Marshall compaction) of mix M25R2.

IT-CY tests were performed by using a servo-pneumatic universal testing machine. On each specimen, the stiffness modulus measurements were performed on two sides of the specimen, by rotating on its axis at 90°. Firstly, for each side measurement, 10 conditioning impulses were applied, followed by five test impulses (3 s repetition time) for which the horizontal stress, horizontal deformation, vertical load and the load-area factor were measured. For a tested specimen of the mix M25R2 in the case of Marshall compaction, an example of a test report is provided in Figure 3.

For each test impulse, a measured stiffness modulus was calculated. A final mean value between these measured stiffness results was determined and reported only if the differences between them were between −20% … +10% of the mean value.

A similar procedure was applied in order to calculate the adjusted stiffness modulus. In this case, the measured values of the stiffness modulus were adapted to the load-area factor with a value of 0.60.

Finally, for each type of bituminous mixture/each type of compaction/each test temperature, a mean value between the results obtained for three specimens was determined and reported in Section 3 for the measured stiffness modulus and the adjusted one.

### 2.6. ITS Test

The determination of the indirect tensile strength of all considered bituminous mixtures was performed with respect to the test procedures presented in SR EN 12697-23 [55] for the determination of ITS and SR EN 12697-12 [56] for the determination of the water sensitivity of all mixes.

According to SR EN 12697-12 [56], the water sensitivity of a bituminous mixture should be checked in the lab on cylindrical specimens compacted at 40 gyrations by using the gyratory press, or 25 blows/side by using the Marshall compaction, or cored samples from slabs compacted with a pneumatic tire compactor at 12 passes. Therefore, compared to the standard test procedure, some changes were made in this study due to the fact that the specimens were compacted differently: Marshall compaction with 50 blows/side, gyratory compaction at 80 gyrations, and cored samples from slabs compacted in the lab by using the same energy value, 39 passes.

Two batches of test specimens were considered: one batch which was conditioned in ‘dry’ conditions at ambient temperature, and the other one the ‘wet’ batch, which was conditioned in water.

The ITS test was performed at a test temperature of 20 °C by applying a compression load diametrically in the direction of the specimen height with a constant speed of 50 mm/min until failure.

For six cylindrical specimens produced as described in Section 2.3 for each type of mixture/each compaction type, the SSD bulk density and dimensions were first determined. Based on these results, the two batches of specimens, dry and wet, were established. Three samples were selected for each batch based on the following criteria: the average length and the average bulk density of the batch should not differ from the individual measurements by more than 3 mm and 10 kg/m^3^, respectively. The average values between the two batches of specimens, dry and wet, did not differ by more than 5 mm and 15 kg/m^3^.

Each group of samples, dry and wet, was conditioned separately. The dry batches were kept at ambient temperature for 72 h. The wet batches were kept in a water bath at 40 °C for the same period. After conditioning, all specimens were brought to the test temperature of 20 °C, for 1 h.

The indirect tensile strength of each tested specimen was calculated by using the equations from SR EN 12697-23 [55]. For each batch, dry and wet, of each type of bituminous mixture/each type of compaction, a mean value between the three tested specimens was reported. Also, the water sensitivity was calculated as the ratio between the indirect tensile strength (ITSR) values obtained for the wet batch and the dry batch. All results are presented in Section 3.3.

## 3. Results and Discussion

### 3.1. Volumetric Measurements

As presented in the experimental plan, first, hydrostatic measurements were performed on three batches of samples for each type of bituminous mixture. All results are reported in Table 2 for the Marshall samples, Table 3 for the cored samples and Table 4 for the gyratory samples.

In the case of the Marshall samples (Table 2), it was observed that the use of the RAP material without rejuvenator leads to an increase in the mix bulk density, water absorption, void content and VMA values. An opposite effect was observed in the case of the VFB results. These trends proved that the aggregates from the RAP material had a higher density, and as the RAP binder was a hard-aged binder, the blend with the fresh binder corresponded to a binder with lower penetration [22,24]. Therefore, the workability and the compaction of the mixes produced with 25% and 50% RAP material without rejuvenator were impacted unfavorably.

As expected, the use of the rejuvenator and the increase in its dose in the production of the bituminous mixtures containing 25% and 50% RAP material led to the reverse effect. The values for the bulk density, water absorption, void content and VMA were decreasing with the increase in rejuvenator content. Thus, a reverse effect could be observed in the case of the VFB values.

Starting from some previous studies [20,22,23,24] where the behavior of the corresponding binder blends of the bituminous mixtures was investigated, it was highlighted that this type of rejuvenator counterbalances the effect of the hard-aged RAP binder, by increasing the penetration values and decreasing the softening point temperature values of the binder blends. Therefore, the use of the rejuvenator led to an improvement in the mix workability. As the binder blends become softer with the increase in the rejuvenator dose, an important part the voids from the mix will be filled with bitumen as VFB values increase with rejuvenator content, an effect that leads to a decrease in void content ($V_{c}$) and finally a decrease in the water absorption values ($W_{a}$).

Similar tendencies could be observed with the cored samples and gyratory samples in the case of samples compacted by using the impact compactor (Marshall compaction). All results are reported in Table 3 and Table 4.

On the other hand, as expected, the compaction mode of the bituminous mixture specimen has a significant impact on its volumetric characteristics.

Regarding the bulk SSD density values, it was observed that higher values were obtained in the case of the impact Marshall compaction, independently of the bituminous mixture type. However, this increase was not very significant—an increase of less than 3% of the bulk density of the Marshall compaction samples. The highest increase was nearly 4.80% of the bulk density of the Marshall compaction samples, in the case of M25R2.

Some differences were observed in the bulk density between the samples produced by using gyratory compaction and the cored samples from the slabs compacted by using the roller compactor. For all tested mixes, a higher value of bulk density was observed in the case of the gyratory specimens. A maximum 3.10% increase was observed in the values obtained for the gyratory compaction, with one exception, M25R2.

Similar observations could be drawn for the results obtained for water absorption: higher values were obtained in the case of the impact Marshall compaction, with one single exception for M25R3—cored samples. For the water absorption results, the increase rates were always higher by 5%, and the maximum increase was approximately 40% for the results obtained for the Marshall compacted specimens (M50R1—cored specimens).

The Romanian norm AND 605, related to the design, production and laying technical conditions for different types of hot mix asphalt, specifies some restrictions related to the water absorption values, which should be between 1.50% vol. and 5% vol. These restrictions are imposed in the case of a conventional HMA (no RAP material, no rejuvenator) with the maximum aggregate size of 16 mm, and the water absorption should be checked on cylindrical Marshall samples.

As can be observed in Table 2, all the obtained results for the water absorption were always lower than 2% vol., and the limitations imposed by the norm were fulfilled in the case of the mixes produced without rejuvenator and for M50R1, M50R2. This was not observed in the cases of the cored and gyratory specimens. For the cored specimens, only the mix M50R0, and for the gyratory specimens, only M25R0 and M50R0, complied with the norm limitations. However, as already mentioned, the norm AND 605 applies only in the case of conventional HMAs.

For the void content results, higher values were obtained in the case of the cored specimens, independently of the bituminous mixture type. These results were always over 36% higher than the results obtained for the Marshall samples. In this case, the highest increase was obtained for the mix M25R3, of approximately 71%. Similar observation could be drawn in the case of the gyratory samples, for which the results obtained for the cored specimens were always higher, by 17%, than those obtained for the Marshall samples, with one exception—M0R0, for which the void content value was higher by only 1.80%.

The same Romanian norm AND 605 specifies some requirements related to the maximum values of void content for a conventional HMA, of 5% (in the case of a road—technical class I–II) and 6% (in case of a road—technical class III–IV), respectively. These requirements should be checked only on cylindrical samples compacted at 80 gyrations. Therefore, as can be seen in Table 4, all obtained results for all mixes fulfilled the mentioned requirements.

From all the observations drawn to this point, it can be highlighted that the three types of compactions considered in the study lead to a different distribution of aggregate and bitumen spreading in the compacted sample, impacting the volumetric characteristics, hydrostatically measured. For the Marshall samples, as the blows were increased up to 50/side, the air voids in the sample were progressively filled; therefore, the density of these specimens was increasing. On the other hand, by using the gyratory compaction, a uniform distribution of the aggregates could be obtained, and by using this compaction method, a lower air void content could be achieved compared to the roller compactor.

### 3.2. Stiffness Modulus Test Results

The IT-CY tests were performed on all nine types of bituminous mixtures for all three types of compaction and at four temperatures, resulting in 108 values for the stiffness modulus. The measured stiffness modulus and its adjusted values were calculated from the raw measurements obtained from the IT-CY tests. All results are reported in Table 5 for measured stiffness modulus and Table 6 for adjusted stiffness modulus.

The maximum standard deviation in the stiffness modulus results (Table 5 and Table 6) was found in the case of the measured stiffness modulus for the mix M50R1—Marshall compaction, test temperature of 10 °C—with a value of 10.20. Therefore, these low standard deviation values indicate that the results obtained for each sample tended to be very close to the mean/reported values.

**Figure 4:**
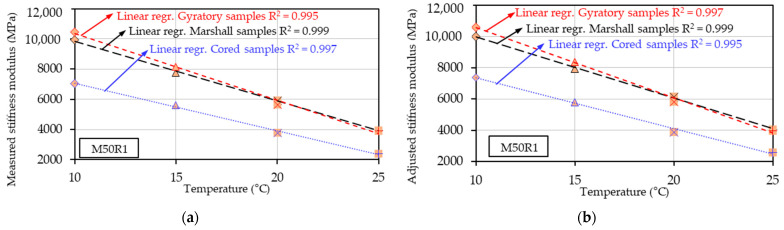
Stiffness modulus results as a function of test temperature for M50R1 mix: (**a**) Measured stiffness modulus; (**b**) Adjusted stiffness modulus.

Also, a relation was observed between the measured and adjusted stiffness modulus values and the test temperature. As an example, Figure 4 shows the results obtained for the mix produced with 50% RAP material and 0.20% rejuvenator (M50R1), where the measured stiffness modulus (Figure 4a) or the adjusted stiffness modulus (Figure 4b) obtained for the three types of compaction were plotted as a function of the test temperature. As expected, for all three types of compaction, the increase in the test temperature corresponded to a linear decrease in the stiffness modulus.

Another important note for the M50R1 mix results is related to the fact that the stiffness modulus values of the Marshall and gyratory specimens were relatively close: higher values for the gyratory specimens for the first two test temperatures and vice versa for higher temperatures (20 °C and 25 °C). The maximum relative difference between these two series of values was approximatively 5.50%. On the other hand, an important decrease (approximatively 33% average value) in $S_{\mathrm{measured}}\left(t\right)$ and $S_{\mathrm{adjusted}}\left(t\right)$ values of the cored specimens compared to those obtained for the other two types of compaction was observed. All these observations are consistent with the results presented above for the volumetric measurements.

Linear regressions were plotted for each type of compaction, and the $R^{2}$ values were calculated. Strong linear relationships were observed between $S_{\mathrm{measured}}\left(t\right)$ or $S_{\mathrm{adjusted}}\left(t\right)$ and the test temperatures, with the $R^{2}$ value always higher than 0.995.

Similar plots could be drawn for each type of mix. However, only the $R^{2}$ values were reported in Table 5 and Table 6 for $S_{\mathrm{measured}}\left(t\right)$ or $S_{\mathrm{adjusted}}\left(t\right)$ values of each mix/each type of compaction. Satisfactory $R^{2}$ values were obtained, always higher than 0.980 in the case of $S_{\mathrm{measured}}\left(t\right)$, $R^{2}$ ≥ 0.978 in the case of $S_{\mathrm{adjusted}}\left(t\right)$. These findings demonstrate a linear decrease in the stiffness modulus values, S(t), as temperature increases, regardless of the type of compaction.

**Figure 5:**
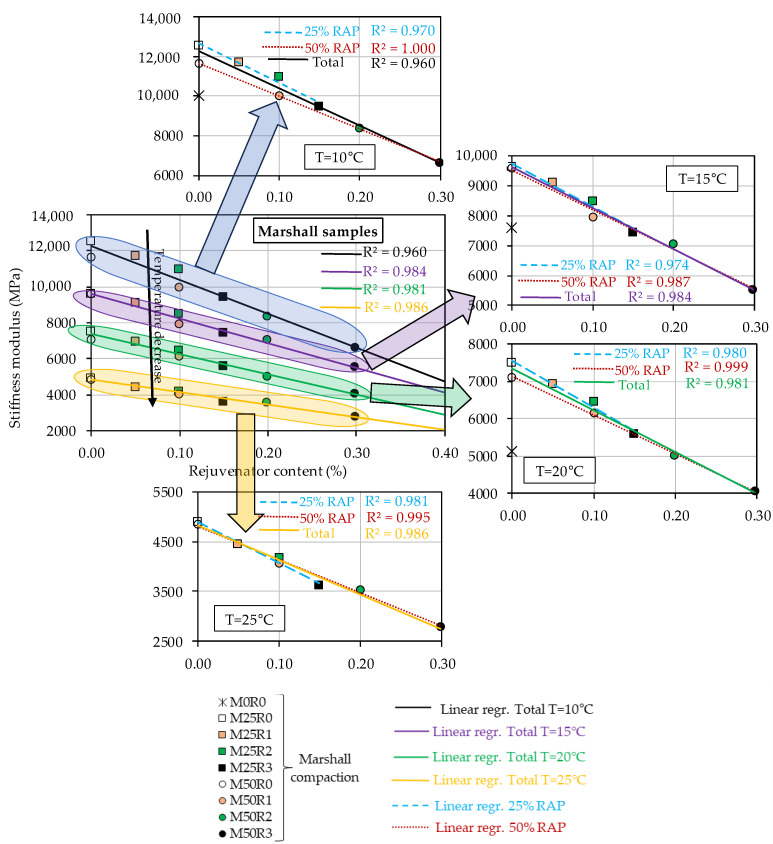
Stiffness moduli at four temperatures as a function of rejuvenator content for all tested bituminous mixtures—Marshall compaction (50 blows/side).

**Figure 6:**
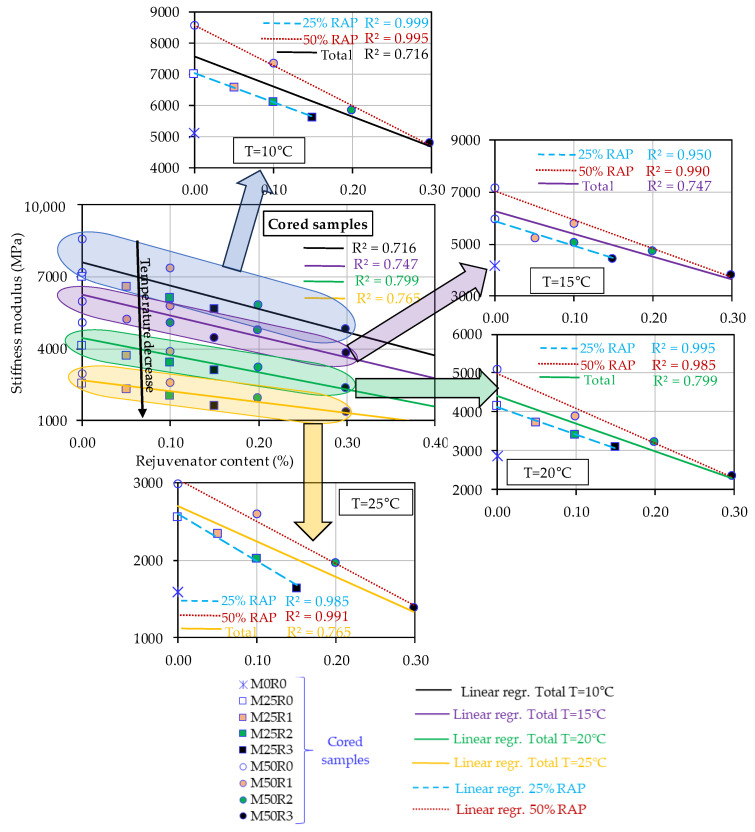
Stiffness moduli at four temperatures as a function of rejuvenator content—cored samples from slabs produced in lab by applying similar compaction energy for all mixes.

**Figure 7:**
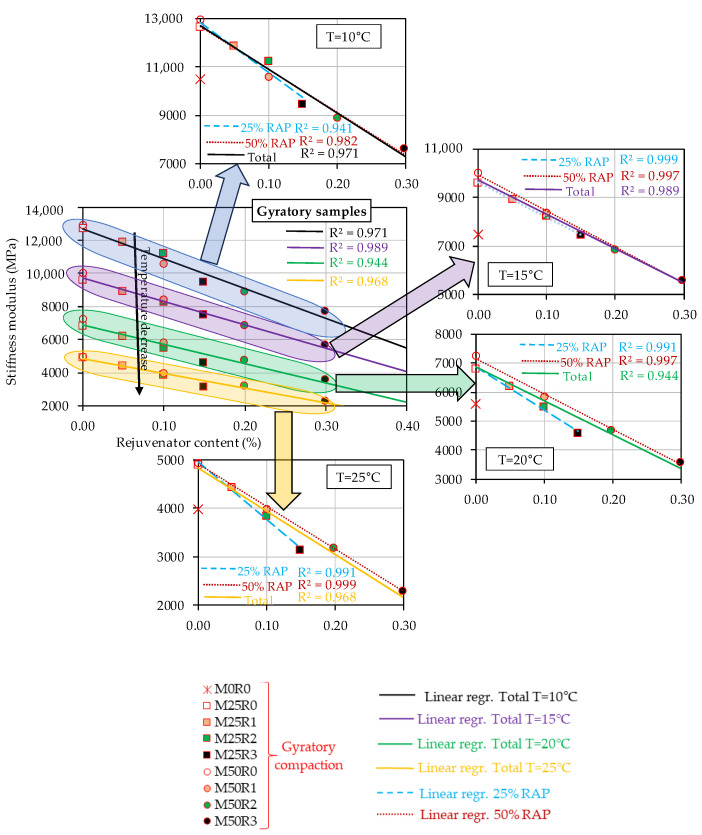
Stiffness moduli at four temperatures as a function of rejuvenator content for bituminous mixtures—gyratory compaction (80 gyrations).

However, the results reported in Table 5 and Table 6 prove that, independently of the compaction mode of the specimens, the increase in the RAP material amount within the mass of the mix leads to an increase in $S\left(t\right)$ values. A reverse effect was observed when the rejuvenator was used. Therefore, all data were plotted as a function of the rejuvenator content by mass of the final mix in order to better study the effect of increasing the RAP and rejuvenator quantities on $S\left(t\right)\;$values. Three graphs were plotted for each compaction type: Figure 5 for Marshall specimens, Figure 6 for cored specimens, and Figure 7 for the gyratory specimens.

These include a general plot in which the stiffness moduli obtained for different test temperatures are plotted against the rejuvenator content and additionally four similar plots for each individual test temperature. For each individual plot, three linear regressions were performed: a general regression for all mixes, a linear regression for mixes produced with 25% RAP material, and another one for mixes produced with 50% RAP material. $R^{2}$ values were calculated for each regression.

In the case of specimens produced by using the impact compaction (Marshall compaction—Figure 5), from the general plot, the use of RAP material without adding a rejuvenator correlates with an increase in stiffness modulus. The use of the bio-rejuvenator leads to a reverse effect or rather a counterbalancing effect on the RAP material. The same observation is valid: the stiffness modulus values decrease linearly with increasing test temperature.

The global linear regressions performed for each series of results obtained at each temperature showed some satisfactory R^2^ values, higher than 0.960. The lowest value of 0.960 was obtained for the tests performed at 10 °C. For the other three series, the R^2^ values were higher than 0.981. Therefore, the results show that the increase in rejuvenator content corresponds to a linear decrease in the stiffness modulus values of all eight mixes produced with RAP material. In the four individual test temperature plots, a better linear relation was observed in the case of the two series for mixes produced with 25% RAP material and another one for mixes produced with 50% RAP material.

In the case of the specimens produced by coring the slabs compacted by using a roller compactor (39 passes), as can be observed in Figure 6, for the general plot, the results were similar to those obtained in the case of the Marshall specimens.

It was observed that for the general (total) linear regressions, some unsatisfactory $R^{2}$ values were obtained, with the highest value of 0.799. Therefore, the other two trends/regressions for the mixes produced with 25% RAP material and for the mixes produced with 50% RAP material were plotted. In the individual plots for the mixes produced with 25% RAP material, some satisfactory $R^{2}$ values higher than 0.950 were obtained independently of the test temperature. This value of 0.950 was obtained in the case of the test temperature of 15 °C; for the other three temperatures, higher values of the coefficient of determination were obtained (higher than 0.985). A similar comment is valid for the mixes produced with 50% RAP material, for which strong $R^{2}$ values higher than 0.985 were obtained.

Possible explanations for these tendencies could include the following:

- the differences between the grading curves of mixtures. As presented in Section 2.2, all mixes were designed by imposing a similar 5.60% total bitumen content (without the rejuvenator) and the same RAP material proportion of 25% RAP 0–8 mm + 75% RAP 8–22.4 mm in order to design a grading curve similar/closer to the one used for the reference mix. However, the use of RAP material leads to a change in the grading curves of the final mixes. Therefore, for the mixes produced with the same RAP material content (25% or 50%), some strong linear relationships with the increase in the rejuvenator content were observed;
- the rejuvenator content was established as a function of the mass of the RAP material; therefore, the total ‘binder’ content (fresh binder + RAP binder + rejuvenator) was increasing with the increase in RAP material. This aspect could impact the compaction process of specimens and the experimental results for the stiffness modulus. Also, the diffusion and dispersion of the rejuvenator in the mass of the mix could be a factor that may cause these trends;
- the differences between the density of the virgin aggregates, the fresh ones, and the RAP aggregates. The mixes with higher concentrations of rejuvenator and RAP material became denser;
- the use of the same compaction energy. This final explanation, combined with all the above possible explanations, could be the reason why the bituminous mixtures produced with similar levels of RAP content presented good linear relations between the stiffness modulus values and the rejuvenator content.

In the case of the specimens produced by using the gyratory compaction, as can be observed in Figure 7, for the general plot, the observations were similar to those made in the case of the Marshall specimens.

The global linear regressions performed for each series of results obtained at each temperature showed satisfactory $R^{2}$ values, higher than 0.944. The lowest value of 0.944 was obtained for the tests performed at 20 °C. Therefore, the general/total linear regressions could be considered valid compared to those obtained in the case of the cored specimens ($R^{2}$ values higher than 0.716).

Linear variations with the rejuvenator content for the mixes produced by using gyratory compaction with the same amount of RAP material of 25% and 50% could be observed for the stiffness moduli obtained for all test temperatures.

For the mixes produced with 25% RAP material, only in case of the test result at 10 °C, a low $R^{2}$ value of 0.941 was obtained. For the other temperatures, some strong relations were obtained, with $R^{2}$ values higher than 0.991. Similar observations can be made for the mixes produced with 50% RAP material, for which some good $R^{2}$ values higher than 0.982 were obtained. The possible explanations for the above-discussed tendencies are similar to those presented for the cored specimens.

The Romanian norm AND 605 specifies some restrictions related to the minimum stiffness modulus value at 20 °C for a conventional HMA (no RAP material, no rejuvenator) with the maximum aggregate size of 16 mm: of 4200 MPa in the case of a road—technical class I–II, and 4000 MPa in case of a road—technical class III–IV. Also, the norm mentions that these requirements should be checked on cylindrical samples produced by using gyratory compaction.

Therefore, as can be seen in Figure 7, in the case of the specimens produced by using gyratory compaction, only the mix M50R3 did not satisfy the minimum conditions imposed by AND 605. This result is due to the fact that this mix contained the highest amount of rejuvenator, which led to a softer behavior. This aspect was confirmed by the behavior of the binder blend corresponding to this mix, which was investigated in previous studies [20,21,22,23,24,25]. However, as already mentioned, the norm AND 605 applies only in the case of conventional HMAs.

For consideration, a similar conclusion is valid for the results presented in Figure 5 for Marshall specimens, for which the same mix M50R3 presented the lowest values of the stiffness modulus. For the cored specimens, a higher stiffness modulus value than the limit was obtained only for M50R0.

Considering the stiffness modulus results presented in Figure 5, it could be concluded that for the test temperature of 20 °C (temperature specified in AND 605), for the mix M50R2, a stiffness modulus value close to the one for the reference mix M0R0 was obtained. In the case of the gyratory specimens (Figure 7), a similar comment is valid for the mixes M50R1 and M25R2. These findings are consistent with the conclusions of previous studies performed on the corresponding binder blends in which the use of 10% rejuvenator by mass of the RAP binder, corresponding to 0.40% rejuvenator by mass of the RAP material, led to a behavior similar/closer to the one observed for the fresh binder, in terms of thermomechanical characteristics [20,21,22,23,24,25,45]. This finding suggests that the use of a proper amount of rejuvenator could lead to a counterbalancing effect on the RAP material, and finally, a new mix can be produced with performance similar/closer to that of a conventional HMA.

All presented results related to the stiffness modulus values obtained at all four temperatures, for all three types of compactions, were consistent with the observations made for the volumetric measurements. The mixes produced with RAP without using the bio-rejuvenator exhibited the highest values for void content, which was linked to an increase in the stiffness modulus values. Conversely, a reverse tendency was obtained with the decrease in the void content values together with the increase in bio-rejuvenator ratios in the mix corresponding to a decrease in the stiffness modulus values independently of the test temperature or the compaction type used.

### 3.3. Indirect Tensile Strength Test Results

The indirect tensile strength tests were performed at 20 °C under two conditions, dry and wet, on all nine bituminous mixtures produced by using three types of compaction (see Section 2.5). For each type of bituminous mixture/each type of compaction, three parameters were investigated: ${\mathrm{ITS}}_{\mathrm{dry}}$, ${\mathrm{ITS}}_{\mathrm{wet}}$ and ITSR. All results are reported in Table 7 and Table 8.

The maximum standard deviation in the ITS results was found in the case of the mix M25R3 for the cored specimens, with a value of 0.033. These low standard deviation values indicated that the ITS results obtained for each sample tended to be very close to the reported (mean) values.

It was shown that the use of the RAP in the production of new bituminous mixtures causes a net increase in the indirect tensile strength (dry or wet) independently of the type of compaction. In the case of the mixes produced without rejuvenator, the ${\mathrm{ITS}}_{\mathrm{dry}}$ results showed an increase of up to 38% for the Marshall samples, 22% for the cored samples, and 32% for the gyratory samples compared to the result obtained for the reference mix (M0R0). For the ${\mathrm{ITS}}_{\mathrm{wet}}$ results, a similar tendency was noted.

The bio-rejuvenator has a counterbalancing impact by decreasing the indirect tensile strength values. Results similar to those obtained in the case of the stiffness modulus values were observed for the samples produced by using gyratory compaction for the mixes produced with 0.40% rejuvenator by mass of the RAP material (M25R2 and M50R2). For these mixes, the ${\mathrm{ITS}}_{\mathrm{dry}/\mathrm{wet}}$ values were comparable/closer (under 5% relative difference) to the ones obtained for the reference mix M0R0. Therefore, the use of a proper amount of rejuvenator could lead to a counterbalancing effect on the RAP material in terms of tensile strength ratios. This observation is consistent with the conclusions of the studies performed on the corresponding binder blends [20,21,22,23,24,25,45].

Regarding the ITSR results, it can be observed that a higher value of 80% was obtained for all samples produced by using gyratory compaction. However, this observation was not valid for the cored samples: for M25R3 and M50R3, the ITSR results were lower than 80%, nor in the case of the Marshall samples, for which the ITSR values were higher than 80% only for M0R0, M25R1 and M50R0. This aspect suggests that the gyratory compaction leads to better water damage resistance (water sensitivity) of the tested bituminous mixtures. Additionally, the increase in the amount of rejuvenator in the mass of the mixes leads to a trend similar to that seen with the ${\mathrm{ITS}}_{\mathrm{dry}/\mathrm{wet}}$ results.

These findings are consistent with the conclusions made regarding the volumetric characteristics. Thus, it can be observed that the decreases in the void content and water absorption values together with the increase in bio-rejuvenator ratios in the mix correspond to a net decrease in ${\mathrm{ITS}}_{\mathrm{dry}}$, ${\mathrm{ITS}}_{\mathrm{wet}}$ and ITSR values independently of the test temperature or the compaction type used.

**Figure 8:**
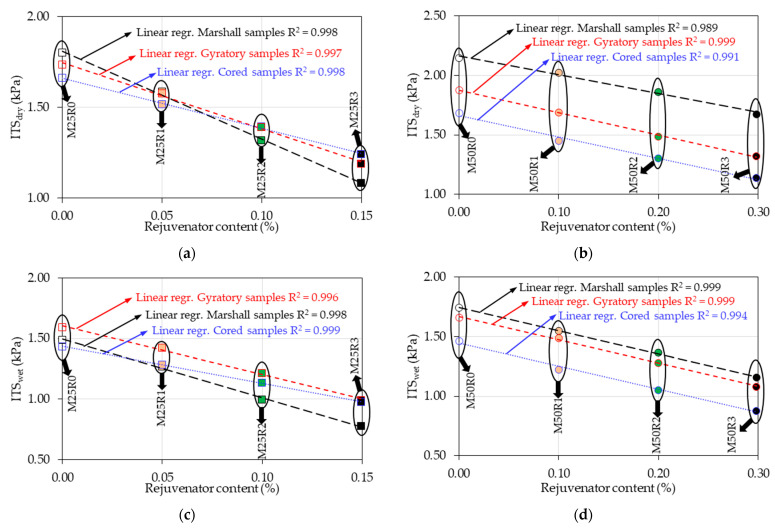
Indirect tensile strengths as a function of rejuvenator content for all bituminous mixtures: (**a**) ${\mathrm{ITS}}_{\mathrm{dry}}$ values for the mixes produced with 25% RAP material; (**b**) ${\mathrm{ITS}}_{\mathrm{dry}}$ values for the mixes produced with 50% RAP material; (**c**) ${\mathrm{ITS}}_{\mathrm{wet}}$ values for the mixes produced with 25% RAP material; (**d**) ${\mathrm{ITS}}_{\mathrm{wet}}$ values for the mixes produced with 50% RAP material.

In Figure 8, ${\mathrm{ITS}}_{\mathrm{dry}/\mathrm{wet}}$ values are shown as a function of the rejuvenator content in order to better explain the rejuvenator’s impact on these outcomes. Some strong linear trends can be observed, regardless of the dry or wet specimens, or the two sample series of 25% RAP material and 50% RAP material. This statement is supported by the high $R^{2}$ values, which were always higher than 0.989.

The possible explanations for the differences in the behavior of the two series of samples produced with 25% RAP material and 50% RAP material and the compaction type used are similar to those presented for the stiffness modulus results in Section 3.2.

### 3.4. Integration of ITS, Penetration vs. Stiffness Moduli Results

The effects of increasing the RAP material and the rejuvenator content together with the use of three distinct compaction methods on the experimental values for the stiffness modulus and indirect tensile strength were highlighted in the previous sections, and it was observed and concluded that all results were consistent. Therefore, in this section, firstly, the correlation between the stiffness modulus and the indirect tensile strength measured at the same test temperature of 20 °C is investigated.

**Figure 9:**
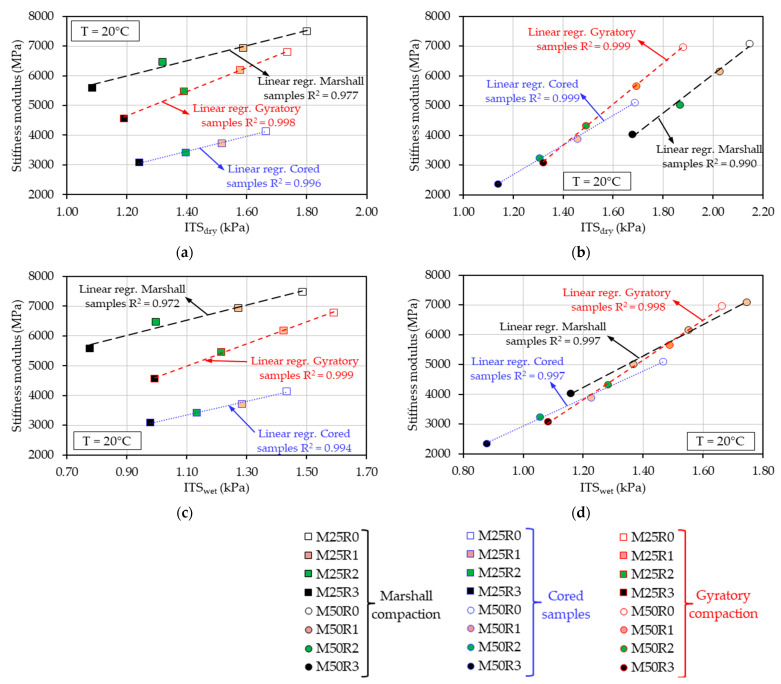
Stiffness moduli as a function of indirect tensile strengths (dry and wet conditions) for bituminous mixtures at 20 °C: (**a**) Stiffness modulus vs. ${\mathrm{ITS}}_{\mathrm{dry}}$ results for the mixes produced with 25% RAP material; (**b**) stiffness modulus vs. ${\mathrm{ITS}}_{\mathrm{dry}}$ results for the mixes produced with 50% RAP material; (**c**) stiffness modulus vs. ${\mathrm{ITS}}_{\mathrm{wet}}$ results for the mixes produced with 25% RAP material; (**d**) stiffness modulus vs. ${\mathrm{ITS}}_{\mathrm{wet}}$ results for the mixes produced with 50% RAP material.

In Figure 9, the results for $S_{\mathrm{measured}}\left(20\;℃\right)$ were plotted as a function of ${\mathrm{ITS}}_{\mathrm{dry}}$ and ${\mathrm{ITS}}_{\mathrm{wet}}$ values for the two series, for mixes produced with 25% RAP material and for mixes produced with 50% RAP material, considering all three types of compaction.

Linear regressions were performed for the mixes prepared with constant RAP material amounts and for each compaction mode. Good linear dependencies between the stiffness modulus and the indirect tensile strength values were found in all of the analyzed cases, supported by $R^{2}$ values that were always higher than 0.972.

However, the compaction mode plays an important role regarding the relations between these two parameters. As can be observed, for the gyratory compaction, the best correlation ($R^{2}$ values always higher than 0.998) was obtained regardless of the analyzed series of mixes or the dry and wet conditions applied for the ITS values. Similarly, for the mixes produced with 25% RAP material and different ratios of bio-rejuvenator compacted with the Marshall impact compactor, the same satisfactory relations between $S_{\mathrm{measured}}\left(20\;℃\right)$ and ${\mathrm{ITS}}_{\mathrm{dry}/\mathrm{wet}}$ values were found, with $R^{2}$ values of 0.977 and 0.972.

The increase in the bio-rejuvenator ratio in the mass of the mix corresponded to a linear decrease in the stiffness modulus and indirect tensile strength results, which were also linearly dependent for the mixes prepared with constant RAP and using the same type of compaction. These results also have a practical implication by taking into consideration that stiffness is related to the elastic capacity of a mix and the strength, which is linked to its permanent deformation and fracture. These are two key parameters used for the analytical design of flexible pavement.

**Figure 10:**
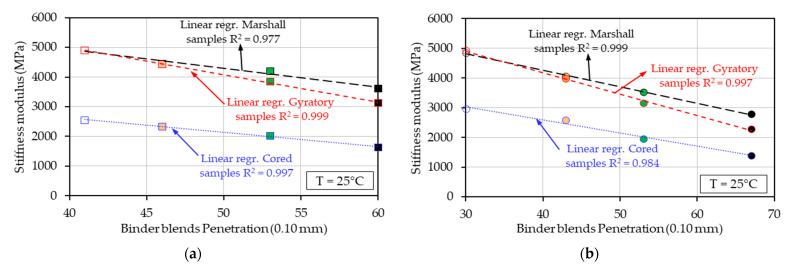
Stiffness moduli of bituminous mixtures at 25 °C as a function of the corresponding binder blend penetration values at 25 °C: (**a**) mixes produced with 25% RAP and their equivalent binder blends; (**b**) mixes produced with 50% RAP and their equivalent binder blends.

Another important result was observed in Figure 10 in the investigation of the possible correlation between the stiffness moduli of the analyzed mixes and the penetration values of the corresponding binder blends. As already mentioned in the previous sections, the corresponding binder blends (RAP binder, 50/70 fresh binder with and without rejuvenator) of the eight tested bituminous mixtures were tested and their behavior investigated in a previous study [22]. Therefore, the penetration values obtained at 25 °C from previous studies were used in this study and plotted as a function of the stiffness modulus test results obtained for the mixes at the same temperature of 25 °C.

In Figure 10, two series of results are considered: Figure 10a—for the results obtained for mixes produced with 25% RAP and their equivalent binder blends, and Figure 10b—for the results obtained for mixes produced with 50% RAP and their equivalent binder blends. In each graph, linear regressions were plotted for the three types of compactions.

A strong linear correlation between the penetration and stiffness modulus values was observed, for which $R^{2}$ values were higher than 0.977. This minimum $R^{2}$ value was obtained for the mixes produced with 25% RAP and distinct ratios of bio-rejuvenator, compacted with the Marshall impact compactor.

An almost perfect linear tendency with $R^{2}$ values higher than 0.997 was obtained in case of gyratory compaction. This observation is consistent with the findings presented in the previous sections. The Marshall and gyratory compaction of mixes produced with 25% RAP material and 50% RAP material gave closer results. For the cored samples, the stiffness modulus values were always lower than the values obtained for the other compactions, with a maximum difference of more than 2000 MPa.

- A global regression for all eight analyzed mixes could not be performed with good results, considering that the use of the RAP led to a shift in the grading curves of the mixes, the use of similar compaction energy for all mixes, and a possible difference between perfect binder blends investigated in previous studies [20,21,22,23,24,25,45] and the blends from the tested bituminous mixtures.

## 4. Conclusions

The conclusions of this study are summarized below based on the testing and analyses presented:

- the use of RAP without bio-rejuvenator in the production of mixes leads to an increase in the volumetric measurements, stiffness modulus and indirect tensile strength values (except for the VFB results) independently of the compaction mode used. The use of the rejuvenator leads to a reverse effect;
- for the M50R2 mix (gyratory compaction), the stiffness modulus and ITS results at 20 °C are comparable/closer (under 5% relative difference) to those obtained for the reference mix. This finding is consistent with the conclusions of previous studies performed on the corresponding binder blends in which the use of 10% rejuvenator by mass of the RAP binder, corresponding to 0.40% rejuvenator by mass of the RAP material, led to a behavior similar/closer to the one observed in the case of fresh binder, in terms of thermomechanical properties. This finding highlights the rejuvenation capacity of this bio-rejuvenator;
- the stiffness modulus and ITS results showed strong linear correlations with the increase in rejuvenator content;
- these results could be explained by several factors: the differences between the grading curves of the mixtures; the rejuvenator content, its diffusion and dispersion; the difference between the density of the virgin aggregates and the RAP aggregates and the use of the same compaction energy;
- for the gyratory compaction, only the M50R3 mix produced with the highest amount of rejuvenator and RAP did not satisfy the minimum conditions imposed by AND 605 (min 4000 MPa for gyratory compaction). This finding was confirmed by the behavior of a binder blend corresponding to this mix investigated in previous studies [20,21,22,23,24,25];
- strong linear relations were observed between $S_{\mathrm{measured}}\left(20\;{}^{\circ}C\right)$ and ${\mathrm{ITS}}_{\mathrm{dry}/\mathrm{wet}}$ values, with $R^{2}$ values higher than 0.972. These results also have a practical implication by taking into consideration that stiffness is related to the elastic capacity of a mix and the strength linked to its permanent deformation and fracture;
- strong linear correlations were observed between $S_{\mathrm{measured}}\left(25\;{}^{\circ}C\right)$ of the mixes and the penetration values of the corresponding binder blends, with $R^{2}$ values higher than 0.977;
- the best correlations between $S_{\mathrm{measured}}\left(20\;{}^{\circ}C\right)$ and ${\mathrm{ITS}}_{\mathrm{dry}/\mathrm{wet}}$ and penetration, with $R^{2}$ values always higher than 0.997, were obtained for the gyratory compaction regardless of the analyzed series of mixes;
- the conclusions of this study are based on the results from the nine tested mixes. Therefore, an investigation on a wider range of materials, including different types and doses of rejuvenators and RAP materials, and a wider experimental campaign that includes the investigation of rutting, fatigue, low-temperature behavior, etc., would be a complementary step that could provide possible directions for improving the modeling and design of recycled hot mix asphalt with rejuvenator.

## Figures and Tables

**Table 1:** Bituminous mixture designs—base material proportions.

Components	Mix Type + Base Material Proportions (%)								
	M0R0	M25R0	M25R1	M25R2	M25R3	M50R0	M50R1	M50R2	M50R3
a. Binder content	5.60	5.60	5.60	5.60	5.60	5.60	5.60	5.60	5.60
● Virgin fresh binder	5.60	4.60	4.60	4.60	4.60	3.60	3.60	3.60	3.60
● RAP binder	-	1.00	1.00	1.00	1.00	2.00	2.00	2.00	2.00
b. Rejuvenator	-	-	0.05	0.10	0.15	-	0.10	0.20	0.30
c. Aggregate content	94.40	94.40	94.35	94.30	94.25	94.40	94.30	94.20	94.10
● Crushed rock	84.96	64.91	64.87	64.84	64.81	44.13	44.09	44.05	44.01
○ 8–16	23.60	16.10	16.09	16.08	16.07	8.02	8.02	8.01	8.00
○ 4–8	20.77	12.94	12.93	12.93	12.92	7.98	7.97	7.96	7.96
○ 0–4	40.59	35.87	35.85	35.83	35.82	28.13	28.10	28.08	28.05
● Natural sand	2.83	0.87	0.87	0.87	0.87	-	-	-	-
● Limestone filler	6.61	4.62	4.62	4.62	4.61	2.27	2.26	2.26	2.26
● RAP aggregates from RAP material 8–22.4	-	18.00	17.99	17.97	17.97	36.00	35.95	35.92	35.88
● RAP aggregates from RAP material 0–8	-	6.00	6.00	6.00	5.99	12.00	12.00	11.97	11.95

**Table 2:** Hydrostatic measurement results—Marshall samples.

Bituminous Mixture	$\rho_{\mathrm{bssd}}$ (kg/m^3^)	$W_{a}$ (% vol.)	$V_{c}$ (%)	VMA (%)	VFB (%)
M0R0	2336	1.55	3.60	16.47	78.17
M25R0	2391	1.68	2.17	15.25	85.76
M25R1	2399	1.44	1.76	15.00	88.28
M25R2	2402	1.31	1.65	15.03	89.02
M25R3	2411	1.03	1.11	14.67	92.45
M50R0	2365	1.88	4.07	16.90	75.90
M50R1	2392	1.70	2.80	16.03	82.52
M50R2	2365	1.56	2.54	16.03	84.13
M50R3	2407	1.18	1.86	15.67	88.10

**Table 3:** Hydrostatic measurement results—cored samples.

Bituminous Mixture	$\rho_{\mathrm{bssd}}$ (kg/m^3^)	$W_{a}$ (% vol.)	$V_{c}$ (%)	VMA (%)	VFB (%)
M0R0	2286	1.13	5.66	18.26	69.10
M25R0	2301	1.31	5.84	18.43	68.38
M25R1	2317	1.13	5.10	17.89	71.51
M25R2	2331	1.03	4.56	17.54	74.05
M25R3	2344	1.04	3.84	17.03	77.45
M50R0	2324	1.83	6.95	19.39	64.19
M50R1	2350	1.21	4.50	17.50	74.37
M50R2	2359	1.16	3.98	17.27	76.94
M50R3	2370	0.95	3.37	16.96	80.17

**Table 4:** Hydrostatic measurement results—gyratory samples.

Bituminous Mixture	$\rho_{\mathrm{bssd}}$ (kg/m^3^)	$W_{a}$ (% vol.)	$V_{c}$ (%)	VMA (%)	VFB (%)
M0R0	2288	1.46	5.56	18.17	69.43
M25R0	2374	1.54	3.37	16.40	79.46
M25R1	2360	1.05	3.33	16.58	79.93
M25R2	2287	0.83	2.85	15.84	82.00
M25R3	2357	0.77	2.85	16.06	82.29
M50R0	2350	1.62	4.68	17.43	73.17
M50R1	2390	1.27	3.72	17.04	78.17
M50R2	2366	1.04	3.31	16.91	80.43
M50R3	2372	0.90	2.88	16.09	82.14

**Table 5:** Measured values of stiffness modulus at four temperatures.

	MEASURED—Stiffness Modulus (MPa)														
Bit. Mix.	Marshall Samples					Cored Samples					Gyratory Samples				
	10 °C	15 °C	20 °C	25 °C	R^2^	10 °C	15 °C	20 °C	25 °C	R^2^	10 °C	15 °C	20 °C	25 °C	R^2^
M0R0	9616	7349	4903	3199	0.995	4913	4063	2719	1443	0.991	10,100	7282	5335	3779	0.982
M25R0	12,184	9336	7259	4743	0.997	6764	5763	3930	2397	0.988	12,298	9267	6490	4658	0.989
M25R1	11,306	8826	6703	4320	0.999	6414	5134	3561	2192	0.999	11,358	8581	5864	4160	0.989
M25R2	10,545	8192	6265	3977	0.999	5944	4989	3236	1879	0.989	10,345	7923	5271	3638	0.991
M25R3	9250	7276	5377	3466	1.000	5330	4313	2916	1513	0.995	9165	7218	4352	2966	0.984
M50R0	11,339	9044	6946	4689	1.000	8253	6897	4833	2807	0.995	12,748	9833	6979	4726	0.997
M50R1	9932	7751	5942	3959	0.999	7047	5609	3767	2380	0.997	10,453	8166	5657	3889	0.997
M50R2	8192	6854	4938	3392	0.996	5570	4476	2983	1671	0.997	8877	6665	4336	2919	0.990
M50R3	6526	5407	3960	2701	0.998	4567	3633	2198	1223	0.993	7436	5253	3084	1983	0.980

**Table 6:** Adjusted values of stiffness modulus at four temperatures.

	ADJUSTED—Stiffness Modulus (MPa)														
Bit. Mix.	Marshall Samples					Cored Samples					Gyratory Samples				
	10 °C	15 °C	20 °C	25 °C	R^2^	10 °C	15 °C	20 °C	25 °C	R^2^	10 °C	15 °C	20 °C	25 °C	R^2^
M0R0	10,000	7584	5113	3380	0.994	5114	4153	2884	1600	0.996	10,492	7495	5589	3974	0.978
M25R0	12,539	9636	7490	4892	0.997	7015	5973	4143	2552	0.989	12,662	9623	6790	4890	0.989
M25R1	11,690	9101	6934	4446	0.999	6596	5243	3724	2336	0.999	11,872	8915	6192	4428	0.988
M25R2	10,963	8480	6466	4188	0.999	6111	5088	3420	2024	0.989	11,233	8235	5473	3837	0.991
M25R3	9453	7448	5584	3620	1.000	5635	4447	3087	1639	0.998	9458	7466	4568	3131	0.985
M50R0	11,643	9604	7099	4849	0.999	8572	7195	5113	2978	0.998	12,936	10,030	7235	4935	0.997
M50R1	10,011	7945	6153	4065	0.997	7382	5794	3896	2590	0.996	10,612	8391	5830	3975	0.999
M50R2	8390	7077	5024	3531	0.993	5858	4749	3253	1960	0.997	8897	6870	4682	3172	0.995
M50R3	6644	5510	4051	2797	0.998	4811	3845	2362	1391	0.992	7662	5612	3584	2295	0.990

**Table 7:** Indirect tensile strength results for all tested bituminous mixtures.

Indirect Tensile Strength Results (kPa)						
Bit. Mix.	Marshall Samples		Cored Samples		Gyratory Samples	
	Dry	Wet	Dry	Wet	Dry	Wet
M0R0	1.556	1.275	1.388	1.166	1.419	1.255
M25R0	1.801	1.486	1.664	1.433	1.735	1.591
M25R1	1.588	1.272	1.516	1.285	1.578	1.423
M25R2	1.319	0.997	1.396	1.134	1.390	1.215
M25R3	1.084	0.776	1.241	0.977	1.190	0.993
M50R0	2.145	1.745	1.686	1.465	1.878	1.662
M50R1	2.026	1.551	1.455	1.227	1.692	1.488
M50R2	1.866	1.368	1.305	1.055	1.489	1.282
M50R3	1.675	1.158	1.139	0.877	1.320	1.081

**Table 8:** Indirect tensile strength ratios (ITSR) for all tested bituminous mixtures.

**Bit. Mix.**	**ITSR (%)**		
	**Marshall Samples**	**Cored Samples**	**Gyratory Samples**
M0R0	81.94	84.01	88.44
M25R0	82.51	86.12	91.70
M25R1	80.10	84.76	90.18
M25R2	75.59	81.23	87.41
M25R3	71.59	78.73	83.45
M50R0	81.35	86.89	88.50
M50R1	76.55	84.33	87.94
M50R2	73.31	80.84	86.10
M50R3	69.13	77.00	81.89

## Data Availability

Data are contained within the article.
